# Supplementary material for: Nanofluidic chips for cryo-EM structure determination from picoliter sample volumes
Source: eLife. 2022 Jan 21;11:e72629. doi: 10.7554/eLife.72629 (PMC8786315; doi:10.7554/eLife.72629)
Supplement: Supplementary file 1. [file elife-72629-supp1.pdf]

Appendix 0 Table 1. Data collection and refinement statistics for collected datasets

	**ApoFtn**<br>**EMD-12901**	**TMV**<br>**EMD-12903**	**T20S**<br>**EMD-12915**
Specimen	3 cryoChips filled with 3.4 mg/mL ApoFtn	2 cryoChips filled with 1.1 mg/mL TMV	1 cryoChip filled with 1.4 mg/mL T20S proteasome
Microscope	TFS Titan Krios	JEOL 3200-FSC	JEOL 3200-FSC
Voltage (kV)	300	300	300
Detector	K2-XP	K2-XP	K2-XP
Energy filter	Gatan BioQuantum	In-column omega filter	In-column omega filter
Number of movies (vitreous/crystalline)	945 (815 / 130)	82 (43 / 39)	121 (91/30)
Pixel size (Å)	0.8127	1.288	1.288
Electron exposure (e^-/Å^2)	63	59.7	59.7
# of frames	90	73	73
Exposure time (s)	9	10.95	10.95
Defocus range (µm)	-1 to -2	-1 to -3.5	-2 to -4
Symmetry imposed	O	helical rotation: 22.036°, helical rise: 1.415 Å	D7
Map sharpening B-factor (Å^2)	-88.3	-65.8	-353.5
Final number of particles / asymmetric units	21,238 / 509,712	14,238 / 284,760	5750 / 80,500
Final map resolution (Å)	3.0	3.7	5.4
Map resolution range (Å)	2.9-3.5	3.5-4.3	5.2-6.5
EMPIAR	10708	10708	10708
