# Supplementary material for: Nanofluidic chips for cryo-EM structure determination from picoliter sample volumes
Source: eLife. 2022 Jan 21;11:e72629. doi: 10.7554/eLife.72629 (PMC8786315; doi:10.7554/eLife.72629)
Supplement: Supplementary file 2. [file elife-72629-supp2.pdf]

Appendix 0 Table 2. Data collection and refinement statistics for reference datasets on holey carbon grids

	**ApoFtn-QF**	**T20S-QF**
Specimen	Quantifoil R2/1 300mesh<br>3.4 mg/mL ApoFtn	Quantifoil R2/1 300mesh<br>1.4 mg/mL T20S
Microscope	TFS Titan Krios	JEOL 3200-FSC
Voltage (kV)	300	300
Detector	K2-XP	K2-XP
Energy filter	Gatan BioQuantum	In-column omega filter
Number of movies	276	879
Pixel size (Å)	0.8127	1.288
Electron exposure (e^-/Å^2)	63	59.7
# of frames	90	93
Exposure time (s)	9	10.95
Defocus range (μm)	-1 to -2	-2 to -4
Symmetry imposed	O	D7
Map sharpening B-factor (Å^2)	-73.6	-175.2
Final number of particles / asymmetric units	21,683 / 520,392	279,332 / 3,910,648
Final map resolution (Å)	2.4	3.2
