# Supplementary material for: Nanofluidic chips for cryo-EM structure determination from picoliter sample volumes
Source: eLife. 2022 Jan 21;11:e72629. doi: 10.7554/eLife.72629 (PMC8786315; doi:10.7554/eLife.72629)
Supplement: Supplementary file 3. [file elife-72629-supp3.pdf]

Appendix 0 Table 3. Model refinement statistics for ApoFtn atomic model.

	**ApoFtn** **7ohf**
Initial model used (PDB code)	2x17
Model resolution (Å, FSC = 0.5)	3.3
CC mask	0.776
**Model composition**	
Nonhydrogen atoms	24 x 1399
Protein residues	24 × 169
**RMSD**	
Bond lengths (Å)	0.008
Bond angles (°)	0.864
**Validation**	
MolProbity score	0.88
Clashscore	1.41
Rotamer outliers (%)	1
**Ramachandran plot**	
Favored (%)	99.4
Allowed (%)	0.6
Disallowed (%)	0
