# Supplementary material for: Nanofluidic chips for cryo-EM structure determination from picoliter sample volumes
Source: eLife. 2022 Jan 21;11:e72629. doi: 10.7554/eLife.72629 (PMC8786315; doi:10.7554/eLife.72629)
Supplement: Supplementary file 4. [file elife-72629-supp4.pdf]

Appendix 0 Table 4. Data and 3D reconstruction parameters of reference datasets

EMPIAR	Protein	Support film	Resolution	B-factor	# particles	Pixel size (Å)	Acq. scheme	Ref.
10200	ApoF	QF-R2/2 holey carbon	1.65 Å	66 $Å^2$ *	426,450	0.814	multi-shot	***Zivanov et al.*** (***2018***)
10272	ApoF	Ultrafoil-R0.6/1 + graphene	2.14 Å	54 $Å^2$ **	41,202	0.649	single-shot	***Naydenova et al.*** (***2019***)
10306	TMV	CF-2/2-2C holey carbon	1.90 Å	41 $Å^2$ **	20,000	0.638	multi-shot	***Weis et al.*** (***2019***)
10389	Urease	QF-R1.2/1.3 holey carbon	1.98 Å	38 $Å^2$ **	119,020	0.639	both	***Righetto et al.*** (***2020***)
10708	ApoFtn	cryoChip SiN_x	2.99 Å	88.3 $Å^2$ **	21,238	0.813	multi-shot	this paper

*B-factor from ResLog plot. **B-factors as determined from Guinier plot.
ApoF: horse spleen apoferritin; ApoFtn: *P. furiosus* apoferritin
